# Ultrasensitive metasurface sensor based on quasi-bound states in the continuum

**DOI:** 10.1515/nanoph-2024-0728

**Published:** 2025-02-12

**Authors:** Ning Li, He Chen, Yunxia Zhao, Yongtian Wang, Zhaoxian Su, Yin Liu, Lingling Huang

**Affiliations:** Beijing Engineering Research Center of Mixed Reality and Advanced Display, 47833 Beijing Institute of Technology, Beijing 100081, China; Beijing Engineering Research Center of Industrial Spectrum Imaging, School of Automation and Electrical Engineering, University of Science and Technology Beijing, Beijing 100083, China

**Keywords:** quasi-bound states in the continuum, all-dielectric metasurface, refractive index sensing, magnetic toroidal dipole, perturbation theory

## Abstract

The quasi-bound state in the continuum (quasi-BIC) of dielectric metasurface provides a crucial platform for sensing, because its almost infinite *Q*-factor can greatly enhance the interactions between light waves and the analytes. In this work, we proposed an ultrasensitive all-dielectric metasurface sensor composed of periodic rectangular amorphous silicon pillars on a quartz substrate. By breaking symmetry of two pillars in unit cell, high *Q* quasi-BIC in the continuous near-infrared band can be excited. The magnetic toroidal dipole (MTD) is demonstrated to play a dominating role in the resonant modes by analyzing near-field distribution and multipole decomposition. The asymmetry degree has a significant impact on sensing performance of the proposed metasurface sensor, whose underlying physical mechanisms is analyzed by perturbation theory. The transmission spectrum and sensing performance of the fabricated metasurface sensor were measured. The experimental results show our designed metasurface sensor not only achieve a high sensitivity of 413/RIU, but also shows a high figure of merit (FOM) of 66 RIU^−1^. This work provides excellent prospects for the excitation of strong MTD resonance quasi-BIC in sensing applications.

## 1 Introduction

Metasurface, as an artificially designed two-dimensional planar material with subwavelength structures, has attracted a lot of attention in recent years due to its remarkable properties of manipulating the characteristics of electromagnetic waves, such as amplitude, phase, and polarization [1]. Metasurface has been widely used in various fields such as filtering [2], absorption [3], invisibility cloaks [4] and sensors [5], [6]. The ultrasensitive and compact sensors are desired widely in various fields [7], [8], [9]. Metasurface based on metal structures can excite surface plasmon resonance, thereby enhancing surface near-field confinement and providing strong optical confinement at sub-wavelength scales, which has been well investigated in sensing [10]. However, due to the large intrinsics associated with metal nanostructures, plasmon resonance is characterized by a low *Q*-factor and a relatively wide bandwidth, which severely limit their sensitivity in optical sensors systems [6]. In recent developments, dielectric metasurfaces which are composed of nano dielectric resonance units featuring high refractive indices, have obtained widespread attention [11], [12], [13]. These dielectric metasurfaces with Mie resonances demonstrate reduced losses and superior diffraction and transmission efficiencies compared to the plasmonic metasurface with metals. Moreover, they are capable of inducing resonances with elevated *Q*-factors and strengthening near-field coupling. These attributes render them an exemplary platform for applications in sensing and surface-enhanced spectroscopy [14], [15], [16]. However, dielectric metasurface sensors confine the electric field within the resonators making peak wavelength less sensitive to changes in the surrounding medium.

Recently, bound state in continuum (BIC) which was first proposed in quantum systems has been realized in nanophotonics, showing almost infinite *Q* factor. BIC is a type of electromagnetic eigenstate that coexists inside the continuous spectrum but completely confined within a resonant system without radiating energy. Ideal BICs exist in the infinitely large periodic system, with the infinitely large *Q* factor, leading to strong interaction between light and matter [17], [18]. According to its radiation suppression mechanism, BIC can be divided into two different types. The first type is symmetric protected BIC, which causes decoupling excitation between resonance modes and radiation waves due to structural symmetry mismatch. This kind of BIC occurs at Γ point in momentum space. The second type is parameter-tuning BIC, which occurs at off-Γ point. This kind of BIC is also regarded as accidental BIC resulting from the destructive interference between different radiative resonance modes, such as Friedrich–Wintgen BIC and Fabry–Pérot BIC [19], [20]. By obliquely incident light sources and breaking structural symmetry in practice, it is possible to weakly couple a symmetric protected BIC with the far-field, leading to a small amount of leakage into the surrounding environment and resulting in finite linewidth and high-*Q* quasi-BIC [21]. Compared to the regular Mie resonances, quasi-BICs are nonlocal resonances, resulted from interaction between adjacent meta-atoms. The metasurface sensors with quasi-BICs can confine the electromagnetic field around the resonator, which can help detect small frequency shifts caused by weak environmental changes in sensing applications and enables the sensitive detection of analytes [22], [23], [24]. Therefore, quasi-BIC has received special attention and been widely studied for ultrasensitive sensing recently.

Leveraging electromagnetic multipole theory as a research instrument to explore fundamental resonances within systems, we are able to attain a more profound comprehension of the properties of BIC [25]. The common electromagnetic multipoles include electric dipole (ED), magnetic dipole (MD), electric quadrupole (EQ), magnetic quadrupole (MQ), toroidal dipole (TD) and so on [26]. It has been found that symmetric protected BIC can be governed by TD resonances. The concept of a toroidal dipole was established in 2010 [27], which has distinct near-field profiles compared ED or MD. By precisely adjusting the nanostructure to achieve TD resonance based on quasi-BIC, high *Q* and field enhancement can be achieved. This property has bright application prospects on sensor. TD can be categorized into electric toroidal dipole (ETD) and magnetic toroidal dipole (MTD) [28], [29], [30]. ETD is composed of as poloidal electric currents flowing on a torus surface and magnetic dipoles arranged in a head-to-tail configuration. Similarly, the MTD is constructed of a closed circle of EDs [31]. Due to the ease with which metasurfaces can excite the moments of ring-shaped MDs, the strong ETD resonance phenomenon has been extensively studied in sensing applications. Song et al. numerically analyzed an all-dielectric hollow metasurface based TD response and MD response, and obtained a maximum sensitivity (*S*) of 160 nm/RIU^−1^ and a maximum figure of merit (FOM) of 575 RIU^−1^ [32]. Wang et al. investigated ultrahigh-*Q* factor TD resonances based symmetric protected BIC in all-dielectric metasurface, and achieved ultrahigh sensitivity level of 489 GHz/RIU [24]. Chen et al. proposed a terahertz metamaterials sensor composed of two pairs of high-index dielectric disks and obtained the sensitivity of 438 GHz/RIU [15]. Although MTD quasi-BIC resonance can achieve high *Q* values and field enhancement, which are very beneficial in the sensing field, there is relatively little research on MTD resonance [33], [34]. This is because realizing a closed circular structure of electric dipoles in nanostructures presents certain challenges. Therefore, further exploration is needed in structural design and mechanism innovation [35], [36], [37].

In this work, we designed ultrasensitive metasurface sensor supported symmetric protected BIC governed by MTD. The MTD quasi-BIC resonance is excited by breaking the symmetry in all-dielectric metasurface composed of periodic nanostructures array, which consist of two pairs of rectangular amorphous silicon pillars. The full wave simulation is adopted to investigate the transmittance and electromagnetic field distribution of the designed metasurface. We analyzed the transformation from BIC modes to quasi-BIC modes by breaking symmetry and verified the quadratic dependence between the asymmetry degree and the radiative *Q*-factor. Moreover, the influence of the incident light angles on the transmission spectrum of the metasurface was also studied. The spatial distribution of electromagnetic fields and the calculated far-field powers scattered by multipoles confirm that the excited mode is MTD resonance. Calculated the influence of the asymmetry degree on sensing performance by perturbation theory and the sensitivity of our designed metasurface has been characterized through simulation to be 547 nm/RIU and the FOM of 237.8 RIU^−1^. Ultimately, the experiment acquired transmission spectrum at different environmental refractive indices to validate the sensing performance of our designed metasurface. This work provides excellent prospects for the excitation of strong MTD resonance quasi-BIC in sensing applications.

## 2 Metasurface structure

**Figure 1:**
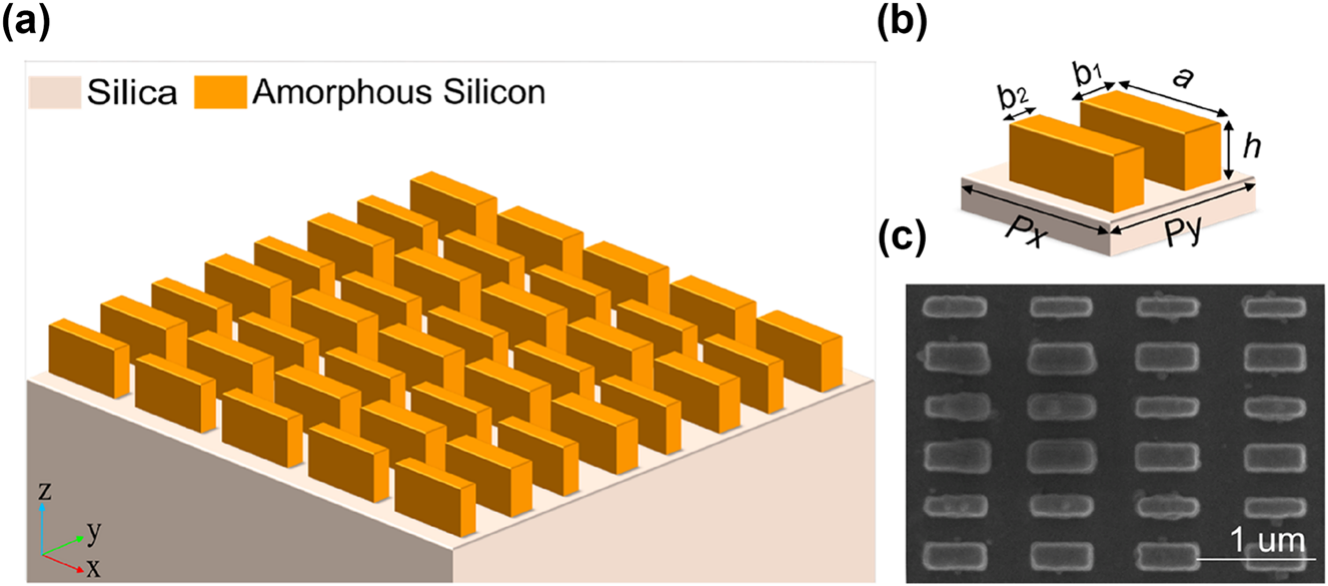
Schematic of the proposed metasurface sensor. (a) Schematic illustration of proposed ultrasensitive metasurface sensor supported symmetric protected BIC governed by MTD. (b) Schematic diagram of a unit cell composed of two rectangular amorphous silicon pillars on a quartz substrate. Specific structural parameters: *Px* = 900 nm, *Py* = 920 nm, *a* = 480 nm, *b*_1_ = 100 nm, *b*_2_ = 140 nm and *h* = 320 nm. (c) The three-dimensional scanning electron microscope image.

**Figure 2:**
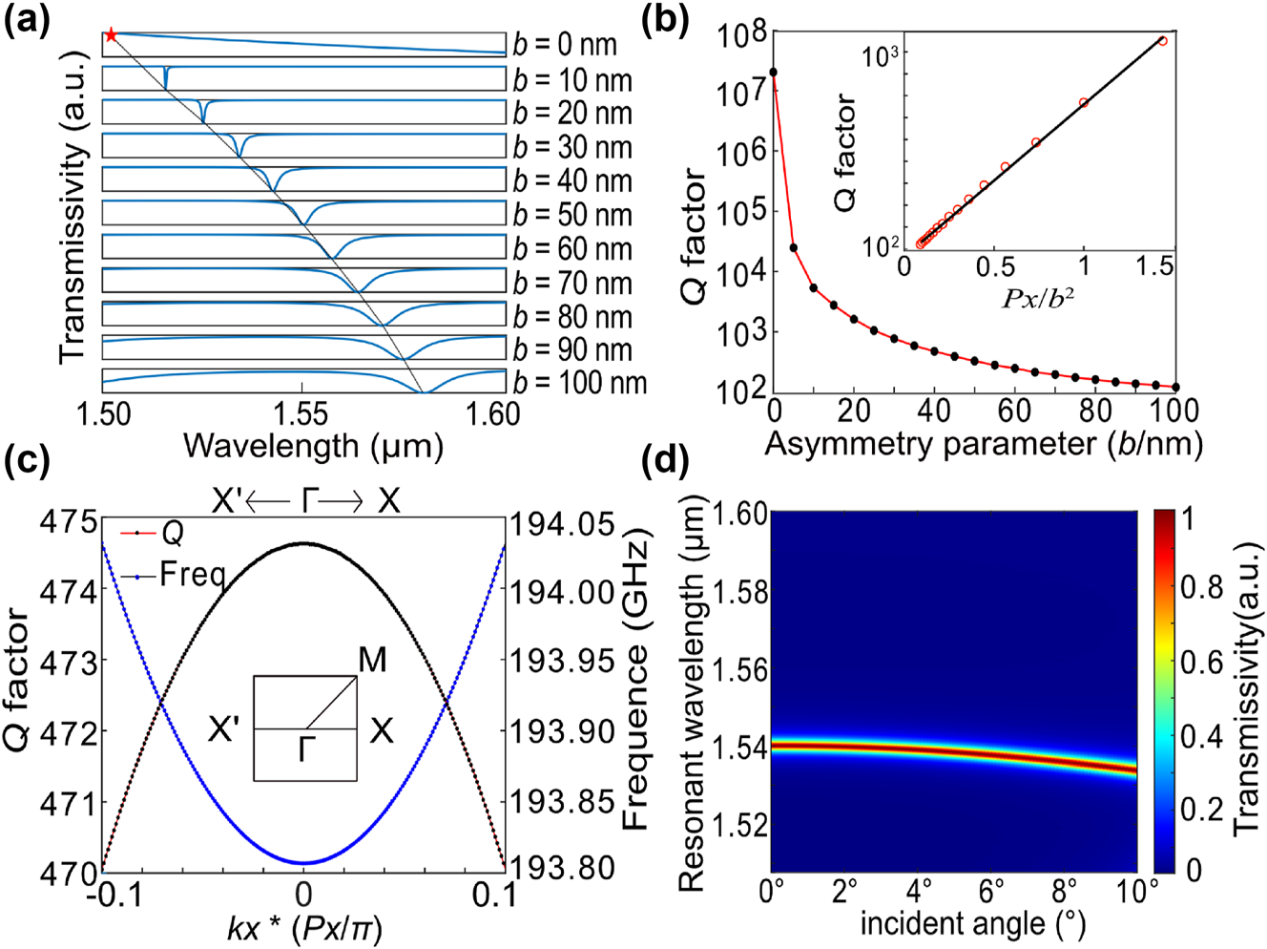
Relation between *Q* factor and asymmetry parameter. (a) Transmission spectrum for asymmetric parameters *b* varying from 0 nm to 100 nm. (b) Relationship between the *Q* factor of the resonance mode and different asymmetric parameters *b*. (c) Band structure and *Q* factor. (d) Schematic of the relationship between incident angle and resonant wavelength.

The schematic illustration of the proposed metasurface sensor is shown in Figure 1a. The unit cell consists of a periodic array of two rectangular amorphous silicon pillars on a quartz substrate, as shown in Figure 1b. The silicon pillars have same heights (*h* = 320 nm) and lengths (*a* = 480 nm), but different widths (*b*_1_ = 100 nm, *b*_2_ = 140 nm). Moreover, *Px* and *Py* are the period of the unit cell in the *x* and *y* direction, respectively. The three-dimensional scanning electron microscope image is shown in Figure 1c. The object wrapped around the structure in Figure 1c is a photoresist, which is caused by poor quality of the developer solution or incomplete cleaning procedures. The C_2_ symmetry of the structure is broken by increasing the width difference between two rectangular pillars to excite the high-*Q* quasi-BIC resonance mode. We define the asymmetric parameter *b* = *b*_2_ − *b*_1_ as the difference in width between two pillars, with its magnitude represents the degree of asymmetry. In order to study the optical properties of the designed metasurface, numerical full wave simulation was conducted. In the simulation, the medium covered the metasurface is water and the refractive index is set as *n* = 1.333. Periodic boundary conditions are applied in both *x*- and *y*-directions and the perfectly matched layer is set to simulate infinite space along the *z*-direction. When the asymmetric parameter *b* is 0, resonance manifests as a perfect BIC that shows there is no energy leakage from the bound state to the free space, as indicated by the red pentagram in Figure 2a. As the asymmetry of the structure increases, we observe that the resonance peak linewidth gradually widened and the resonance wavelength gradually redshifts. When the asymmetric parameter *b* is large, the resonance peak is a pronounced asymmetric Fano linear. A wider line width implies decreasing *Q* factor of the resonance, as shown in Figure 2b. As *b* gradually increases, the *Q* factor sharply decreases, and the illustration also shows that the *Q* factor is inversely proportional to *b* ^2^. Taking the structure with an asymmetric parameter *b* = 40 nm as an example, we computed the band structure and analyzed the effects of oblique incidence on the transmission spectrum. As shown in Figure 2c, it can be observed that the *Q* factor slightly decreases with the increase of *kx*-vector, while the frequency slightly increases with the increase of *kx*-vector in the Γ–X direction. We calculated the transmission spectrum of the metasurface with different tilt angles *θ* ranging from 0° to 10° as shown in Figure 2d, which further shows that as the incident angle increases, the resonant wavelength decreases while the full width at half maximum (FWHM) remains almost unchanged, so the mode is robust in momentum space.

**Figure 3:**
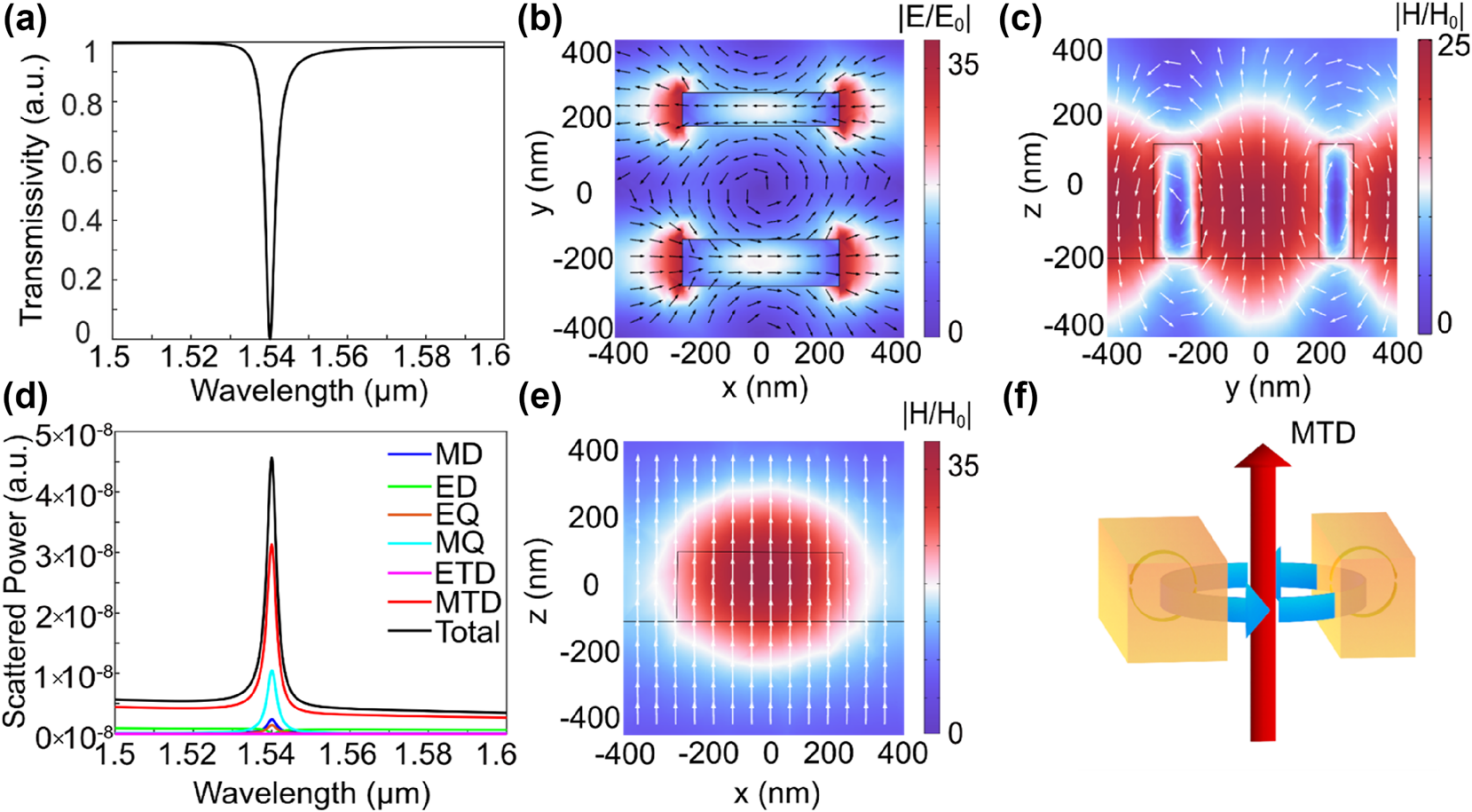
The properties of MTD resonance. (a) The transmission spectrum when *x*-polarized incident is irradiated on the designed metasurface. (b) The electric field and displacement current distributions at the resonance wavelength of *x*–*y* plane. (c) and (e) The magnetic field and magnetic vector distribution at the resonant wavelength in the *y*–*z* (c) and *x*–*z* planes (e), respectively. (d) The total scattered power and the contribution of different multipoles. (f) Schematic of the MTD in the designed metasurface.

Then, we calculate the transmission spectrum, the near-field distribution and far-field radiation when *x*-polarized incident along the *z*-axis is irradiated on the metasurface with asymmetric parameter *b* = 40 nm. Figure 3a show the transmission spectrum of the designed metasurface. It can be seen that the resonance wavelength is located at 1,541.2 nm, and the resonance shows a significant asymmetric Fano-type features with the FWHM is 2.3 nm. In order to study the characteristics of resonance, electromagnetic multipole decomposition in Cartesian coordinates is used to calculate the contributions of various multipole components to far-field radiation based on the induced current [38]. Cartesian multipole moments can be expressed by using the induced currents $\vec{j}$ as

Electric dipole moment:

$$\vec{P}=\frac{1}{i\omega }\int \vec{j}{\text{d}}^{3}r$$

Electric toroidal dipole moment:

$$\vec{T}=\frac{1}{10c}\int \left[\left(\vec{r}\cdot \vec{j}\right)\vec{r}-2r^{2}\vec{j}\right]{\text{d}}^{3}r$$

Magnetic dipole moment:

$$\vec{M}=\frac{1}{2c}\int \left(\vec{r}\times \vec{j}\right){\text{d}}^{3}r$$

Magnetic toroidal dipole moment:

$$\vec{G}=\frac{k^{2}}{20}\int {\vec{r}}^{2}\left(\vec{r}\times \vec{j}\right){\text{d}}^{3}r$$

Electric quadrupole moment:

$$\vec{Q_{\alpha \beta }^{\left(e\right)}}=\frac{1}{2i\omega }\int \left[r_{\alpha }j_{\beta }+r_{\beta }j_{\alpha }-\frac{2}{3}\left(\vec{r}\cdot \vec{j}\right)\delta_{\alpha ,\beta }\right]{\text{d}}^{3}r$$

Magnetic quadrupole moment:

$$\vec{Q_{\alpha \beta }^{\left(m\right)}}=\frac{1}{3c}\int \left[{\left(\vec{r}\times \vec{j}\right)}_{\alpha }r_{\beta }+{\left(\vec{r}\times \vec{j}\right)}_{\beta }r_{\alpha }\right]{\text{d}}^{3}r$$

where *ω* denotes the angular frequency, $\vec{r}$ is the position vector, *k* is the wavevector, *c* is the speed of light and *α*, *β* = *x*, *y*, *z*. Here, we ignore the small contribution of higher-order multipoles. The scatted powers of these multipole moments are calculated from:

$$I_{p}=\frac{2\omega^{4}}{3c^{3}}{\left|\vec{P}\right|}^{2},I_{M}=\frac{2\omega^{4}}{3c^{3}}{\left|\vec{M}\right|}^{2},I_{T}=\frac{2\omega^{6}}{3c^{5}}{\left|\vec{T}\right|}^{2},I_{G}=\frac{2\omega^{6}}{3c^{5}}{\left|\vec{G}\right|}^{2},$$

$$I_{Q^{\left(e\right)}}=\frac{\omega^{6}}{5c^{5}}{\left|\vec{Q_{\alpha \beta }^{\left(e\right)}}\right|}^{2},I_{Q^{\left(m\right)}}=\frac{\omega^{6}}{40c^{5}}{\left|\vec{Q_{\alpha \beta }^{\left(m\right)}}\right|}^{2}$$

The total scattered power of the multipole moments can be given as:

$$I_{\text{total}}=I_{P}+I_{M}+\frac{4\omega^{5}}{3c^{4}}\left(\vec{P}\cdot \vec{T}\right)+I_{T}+\frac{4\omega^{5}}{3c^{4}}\left(\vec{M}\cdot \vec{G}\right)+I_{G}+I_{Q^{\left(e\right)}}+I_{Q^{\left(m\right)}}$$

where $\frac{4\omega^{5}}{3c^{4}}\left(\vec{P}\cdot \vec{T}\right)$ and $\frac{4\omega^{5}}{3c^{4}}\left(\vec{M}\cdot \vec{G}\right)$ are the interference terms of the ED and ETD, MD and MTD.

From Figure 3d, it can be observed that the MTD dominates this resonance. Meanwhile, the electromagnetic field response at the resonance wavelength can further verify that the MTD resonance is the dominant response and the black arrow and white arrow represent current and magnetic vector, respectively. As shown in Figure 3c, two opposite circular directions magnetic field vector distributions in the *y*–*z* plane at *x* = 225 nm can excite two EDs in different directions along the *x* axis. In combination with the incident light, MTD response along the *z*-axis is then produced by the circular ED in the *x*–*y* plane at *z* = 0 nm, as shown in Figure 3b. Figure 3e shows the distribution of magnetic field and magnetic vector in the *x*–*z* plane at *y* = 0 nm. Figure 3f further intuitively displays the field distribution during resonance. It can be seen that the MTD response significantly enhances the field distribution, and the distribution of the electric and magnetic fields reveals that the enhanced field primarily concentrates at the edges of the structure, which is highly advantageous for sensing.

**Figure 4:**
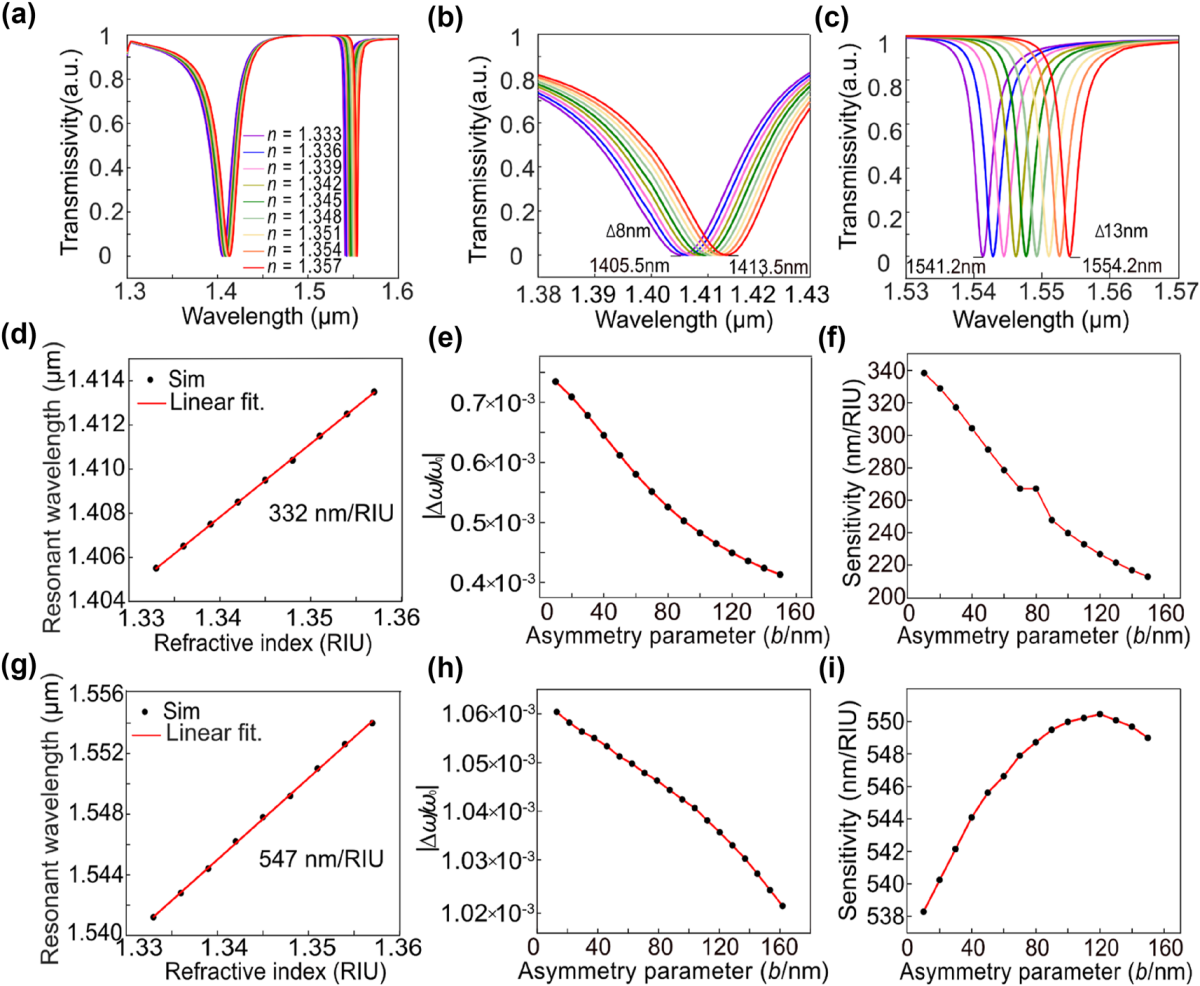
Sensitivity analysis of the designed BIC metasurface. (a) Simulated transmission spectrum for different environmental refractive indices. (b) and (c) Simulated transmission spectrum for dipole resonance (b) and q-BIC resonance (c), respectively. (d) and (g) The relationship between resonant wavelength and environmental refractive index for dipole resonance (d) and quasi-BIC resonance (g). (e) and (h) The relationship between the relative resonance frequency shift and asymmetric parameters *b* for dipole resonance (e) and quasi-BIC resonance (h), respectively. (f) and (i) The refractive index sensitivity theoretically calculated under different asymmetry parameters *b* for dipole resonance (f) and quasi-BIC resonance (i).

We then simulate the resonance peak shifts at different environmental refractive indices and evaluate the refractometric index sensitivities, which is defined as the ratio of the resonant wavelength shift $\Delta \lambda \left(\text{nm}\right)$ to the variation of the surrounding refractive index $\Delta n\left(\text{RIU}\right)$ . Figure 4a shows the resonance spectrum of the metasurface in different environmental refractive indices. In addition to the quasi-BIC resonance with the FWHM of 2.3 nm we are focusing on, there exists a low-*Q* Dipole resonance at shorter wavelengths and the FWHM is 27.2 nm. As environmental refractive index varies, the peak wavelengths of the quasi-BIC and Dipole resonances exhibit shifts of 8 nm and 13 nm, respectively, as shown in Figure 4b and c. By linearly fitting the environmental refractive index with the corresponding resonance peak wavelength to obtain the sensitivity of 547 nm/RIU for the MTD resonance based on quasi-BIC, and the sensitivity of 332 nm/RIU for the Dipole resonance as shown in Figure 4d and g. The calculated FOM value which is defined as the ratio of the refractive index sensitivity to the FWHM of corresponding resonance is 237 RIU^−1^, 12 RIU^−1^. The sensitivity of MTD resonance based on quasi-BIC is significantly higher than that of Dipole resonance.

To verify whether the sensitivity of the quasi-BIC resonance is always higher than that of the Dipole resonance, we have used perturbation theory to calculate the sensitivity of two types of resonances under different asymmetric parameters *b*. According to perturbation theory, the relative resonance frequency shift $\left|\frac{\Delta \omega }{\omega_{0}}\right|$ is determined by the ratio of the variation of the electromagnetic energy in the perturbed area and the total electromagnetic energy of the system [39]:

$$\begin{array}{ll} \left|\frac{\Delta \omega }{\omega_{0}}\right| & =\frac{\int \left|\Delta \varepsilon \right|{\left|E\left(r,\omega_{0}\right)\right|}^{2}\text{d}V_{\text{out}}}{\int \varepsilon {\left|E\left(r,\omega_{0}\right)\right|}^{2}\;+\;\mu {\left|H\left(r,\omega_{0}\right)\right|}^{2}\text{d}V_{\text{all}}}\\ & =\left|\Delta \varepsilon \right|\cdot \frac{\int {\left|E\left(r,\omega_{0}\right)\right|}^{2}\text{d}V_{\text{out}}}{\int \varepsilon {\left|E\left(r,\omega_{0}\right)\right|}^{2}\;+\;\mu {\left|H\left(r,\omega_{0}\right)\right|}^{2}\text{d}V_{\text{all}}} \end{array}$$

where, *ω*_0_ is the initial resonance frequency, Δ*ω* is the resonance shift, Δ*ɛ* represents the change of local dielectric constant caused by the variation of the surrounding medium of the resonator. *ɛ* and *μ* are permittivity and permeability, respectively. $E\left(r,\omega_{0}\right)$ and $H\left(r,\omega_{0}\right)$ are the electric field and magnetic field at the resonant frequency, respectively. The outside volume *V*_out_ is defined by a volume of *P*_*x*_ × *P*_*y*_ × 500 nm^3^, where the height of 500 nm includes the resonator height (320 nm) and a volume above the resonator top surface with a height of 180 nm, minus the resonator volume. *V*_all_ represents a cube with a volume of *P*_*x*_ × *P*_*y*_ × 1 μm^3^, covering the near-field region. The above equation can be divided into two terms, as the dielectric constant is isotropic. The first term Δ*ɛ* represents the change of the dielectric constant due to the variation of environmental refractive index. The second term is the relative change in the distribution of electric field energy, which is determined by asymmetry parameter *b* and calculated through the distribution of electromagnetic fields in the near-field. When the environmental refractive index *n* changes from 1.333 to 1.336, that is, when the change of permittivity is constant, the relative resonance frequency shift was calculated. Figure 4e and h shows the relationship between relative resonance frequency shift and asymmetric parameters *b* for the Dipole resonance and the quasi-BIC resonance, respectively. The relative resonance frequency shift decreases with the increase of asymmetric parameters. The sensitivity of quasi-BIC and Dipole resonance was calculated based on the resonance frequency shift and resonance frequency. Figure 4f shows the sensitivity of the Dipole resonance consistently decreases with the increase of the asymmetry parameter *b* and remains always lower than the sensitivity of the quasi-BIC resonance. The sensitivity of the quasi-BIC resonance initially increases with the increment of the asymmetry parameter *b*, reaching its maximum sensitivity 550.48 nm/RIU when *b* equals 120 nm, after which the sensitivity exhibits a declining trend as shown in Figure 4i.

**Figure 5:**
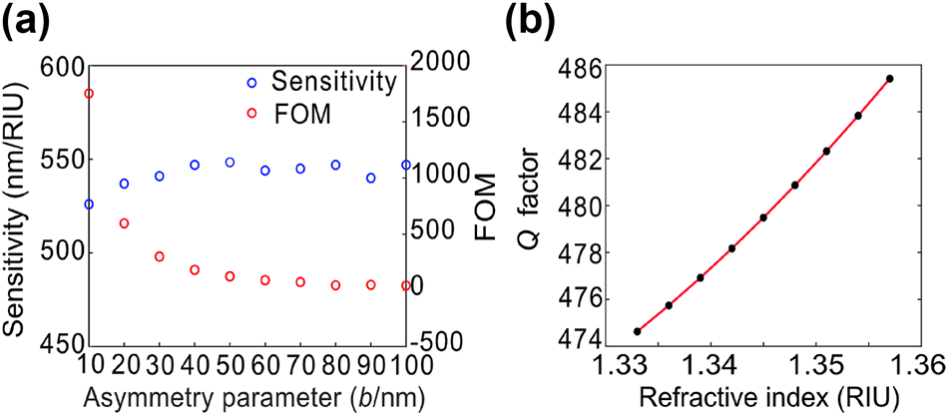
Sensitivity and *Q* factor depend on environmental refractive index. (a) The trends of simulated refractive index sensitivity and figure of merit (FOM) with respect to the variation of the asymmetry parameter. (b) The relationship between *Q* factor and environmental refractive index.

Due to the fact that the sensitivity of quasi-BIC resonance is always higher than that of Dipole resonance, we only focus on quasi-BIC resonance. We calculated the dependence of refractive index sensitivity and FOM on different asymmetric parameters *b* through full wave simulation, as shown in Figure 5a. The refractive index sensitivity of quasi-BIC mode is easily affected by different asymmetric parameters. Similar to the results of perturbation theory, the refractive index sensitivity increases first and then decreases with the increase of asymmetric parameters. When the asymmetric parameter *b* = 40 nm, the refractive index sensitivity reaches saturation. However, FOM decreases with the increase of asymmetric parameters. Therefore, we set the asymmetric parameter *b* = 40 nm to minimize the decrement of the FOM. For the structure with asymmetric parameter *b* = 40 nm, the *Q* factor varies slightly under different environmental refractive indices, as shown in Figure 5b. As the refractive index difference between the quartz substrate and the environment gradually decreases, the *Q* factor value increases continuously with the increase of environmental refractive index.

**Figure 6:**
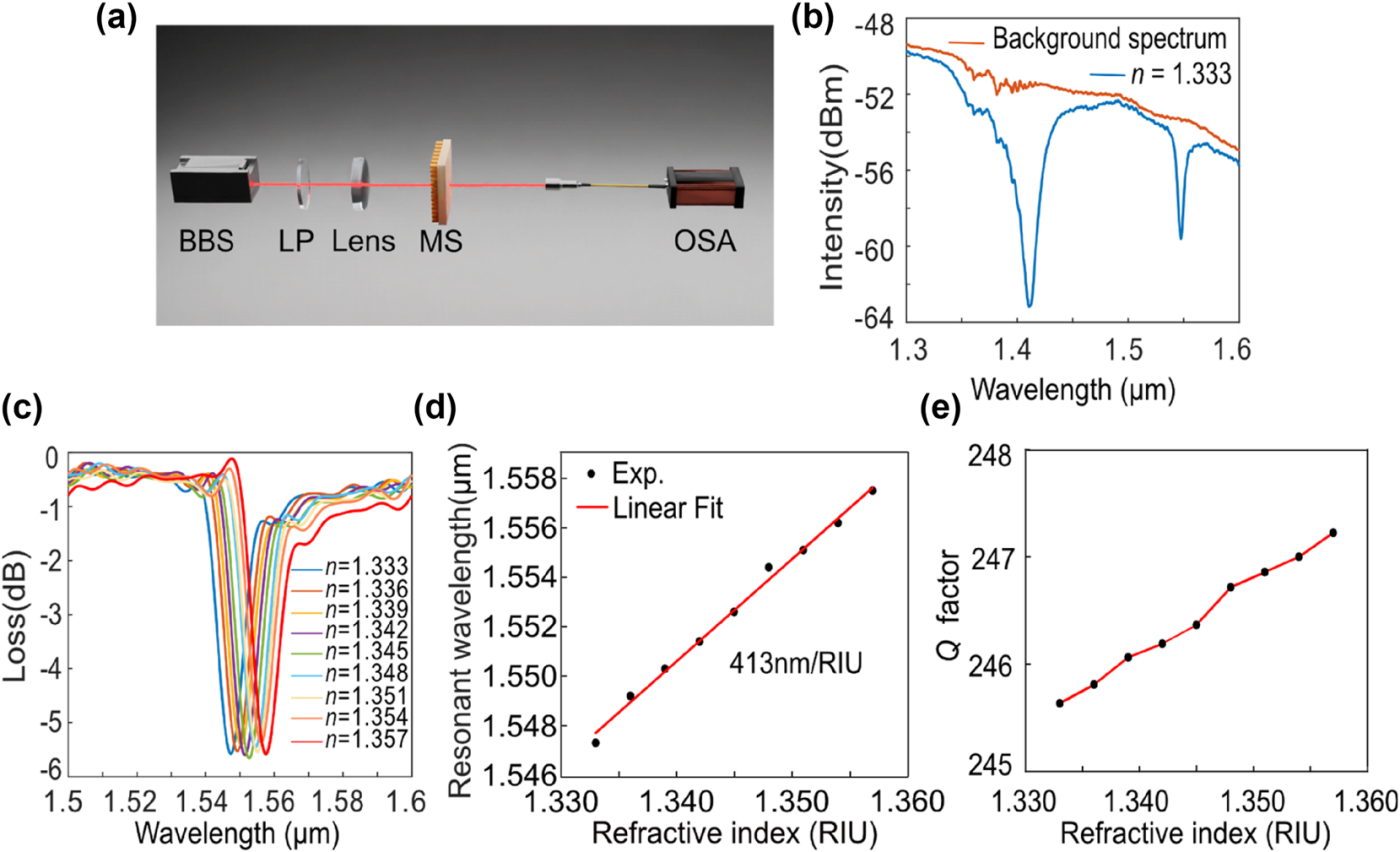
Measurement results of the refractive index sensitivity. (a) Schematic diagram of the measurement system. (b) Background spectrum and transmission spectrum in pure water. (c) Experimental measurement of transmission spectra with different environmental refractive indices. (d) The resonant wavelength as a function of the environmental refractive index. The refractive index sensitivity is determined from the slope of the linear fitting curve. (e) The variation of experimentally measured *Q* factors for the resonances as the refractive index changes.

To evaluate the sensing performance of the fabricated metasurface, we have first quantified the refractive index sensing capabilities of the metasurface. The transmission spectrum was measured to characterize the sensing performance of the fabricated sample. Figure 6a depicts the experimental setup. The light source from the broadband laser source (NKT Photonics SuperK COMPACT) is focused onto the fabricated metasurface using a lens and then collected using an Optical Spectrum Analyzer (Yokogawa AQ6370C) through a fiber coupler. Since the fabricated metasurface is polarization dependent, we add a linear polarizer between the source and the lens to adjust the polarization of the light. Measure the transmission spectrum of a quartz substrate to take it as the background spectrum. Then, measure the transmission spectrum of the sample with water covering it. In addition to the expected quasi-BIC resonance at long wavelengths, we discovered that resonance at short wavelengths with low *Q* values can also be excited as shown in Figure 6b. However, our focus is solely on the quasi-BIC resonance at long wavelengths. Subsequently, subtract the background spectrum from the water-covered sample spectrum to obtain the transmission spectrum corresponding to an environmental refractive index of 1.333. Repeat this process for different environmental refractive indices to obtain a series of transmission spectra. Figure 6c shows the shift of resonance wavelength with respect to refractive index using NaCl solutions of different concentrations. As the refractive index increased, the resonance wavelength gradually shifted towards the longer wavelength region. The FWHM is calculated as 6.25 nm by selecting the wavelength difference corresponding to the intensity that is 3 dB higher than the minimum intensity in the transmission spectrum. We linearly fit the environment refractive index with the corresponding resonance peak to obtain a sensitivity of 413 nm/RIU and FOM is 66 RIU^−1^ as shown in Figure 6d. Figure 6e illustrates the relationship between environmental refractive index and *Q* factor, with the *Q* factor value slightly increasing as the environmental refractive index increases. Compared with the simulation results, both sensitivity and *Q* value are relatively reduced due to the finite size and fabrication imperfections. As shown in Table 1, after comparing our results with previous works, it can be seen that the proposed structure has better sensing performance and simpler structure, providing a valuable reference for future sensor applications.

**Table 1:** Comparison of the sensitivity value and FOM of dielectric metasurfaces in different studies.

Reference	Phenomenon	Structure	Sensitivity (nm/RIU)	FOM (RIU^−1^)
[40]	TD mode	Hollow	161	80
[41]	Quasi-BIC	Elliptical and cylindrical	305	68
[42]	MD mode	Nanodisk	385	15
[43]	Quasi-BIC	Elliptic nanopillars	122	4.36
[44]	MD mode	Dual nanorod	408	62
This work	MTD mode	Two pillars	413	66

## 3 Conclusions

In conclusion, an all-dielectric amorphous silicon metasurface composed of two rectangular pillars and achieve ultrasensitive sensing via the excitation of magnetic toroidal dipole based on bound states in the continuum. We analyzed the transformation spectrum from BIC modes to quasi-BIC modes by breaking symmetry, a distinct sharp Fano resonance can be observed in 1,541 nm, and the *Q* factor is ∼474. The spatial distribution of electromagnetic fields and the calculated far-field powers scattered by multipoles confirm that the excited mode is MTD resonance, which enhanced electromagnetic field interaction between surrounding medium and nano-structures. The impact of asymmetry degree on sensing performance was analyzed using perturbation theory. The experimental results verified that the sensitivity of our designed metasurface is 413 nm/RIU, and the figure of merit (FOM) is 66 RIU^−1^. The design and scheme of the proposed metasurface are founded on a straightforward and uncomplicated approach, eliminating the necessity for intricate structural geometry and environmental design. This work provides excellent prospects for the excitation of strong MTD resonance quasi-BIC in sensing applications.

## 4 Methods

**Fabrication.** Metasurface were fabricated on quartz substrate by following the steps of patterning, deposition, and lift-off. Initially, plasma-enhanced chemical vapor deposition (PECVD) technology was employed to deposit amorphous silicon thin films onto the quartz substrates. Subsequently, a layer of PMMA positive photoresist was applied on top of these amorphous silicon thin films via spin coating. The sample was then exposed using electron beam lithography (EBL), and the pattern was revealed after the development process. This pattern was subsequently transferred to a 40 nm-thick chromium layer, which was deposited by electron beam evaporation. The PMMA was removed by ultrasonication in acetone at 60 °C, and the final structure was obtained through a reactive ion etching (RIE) process.
